# The Role of Host Cell Glycans on Virus Infectivity: The SARS‐CoV‐2 Case

**DOI:** 10.1002/advs.202201853

**Published:** 2022-11-23

**Authors:** Silvia Acosta‐Gutiérrez, Joseph Buckley, Giuseppe Battaglia

**Affiliations:** ^1^ Institute for the Physics of Living Systems University College London London Central London UK; ^2^ Institute of Structural and Molecular Biology University College London London Central London UK; ^3^ Department of Chemistry University College London 20 Gordon St London Central London WC1H 0AJ UK; ^4^ Institute for Bioengineering of Catalunya (IBEC) The Barcelona Institute of Science and Technology Barcelona Barcelona 08028 Spain; ^5^ Catalan Institution for Research and Advances Studies (ICREA) Barcelona Spain

**Keywords:** glycans, glycocalyx, multiplexing, multivalency, nanoparticles, super‐selectivity, viral infectivity

## Abstract

Glycans are ubiquitously expressed sugars, coating the cell and protein surfaces. They are found on many proteins as either short and branched chains or long chains sticking out from special membrane proteins, known as proteoglycans. This sugar cushion, the glycocalyx, modulates specific interactions and protects the cell. Here it is shown that both the expression of proteoglycans and the glycans expressed on the surface of both the host and virus proteins have a critical role in modulating viral attachment to the cell. A mathematical model using SARS‐Cov‐2 as an archetypical virus to study the glycan role during infection is proposed. It is shown that this occurs via a tug‐of‐war of forces. On one side, the multivalent molecular recognition that viral proteins have toward specific host glycans and receptors. On the other side, the glycan steric repulsion that a virus must overcome to approach such specific receptors. By balancing both interactions, viral tropism can be predicted. In other words, the authors can map out the cells susceptible to virus infection in terms of receptors and proteoglycans compositions.

## Graphical abstract

The authors depict the attachment of the severe acute respiratory syndrome coronavirus 2 (SARS‐Cov‐2) to the cell surface in two steps: (1) Attachment to heparan sulphate and (2) binding to the entry receptors. They show both the SARS‐Cov‐2 virion, with all‐atom structures of the spike glycoproteins and the cell surface process.

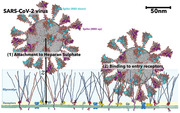

## 1 Introduction

The Coronavirus disease (COVID‐19) pandemic, caused by the severe acute respiratory syndrome coronavirus 2 (SARS‐CoV‐2), has caused over 551 million infections and over 6.3 million deaths worldwide (World Health Organization, WHO). SARS‐CoV‐2 belongs to the beta coronavirus genus part of the c*oronaviridae* family. The same family as the middle east respiratory syndrome coronavirus (MERS‐CoV) and the severe Acute Respiratory Syndrome coronavirus (SARS‐CoV), two well‐established pathogens which emerged in the middle east in 2012 and the south of China in 2003, respectively.^[^ ^1^ ^]^ Coronaviruses have a high zoonotic potential, as demonstrated by recent and past outbreaks. Therefore, understanding their mechanism of infection is paramount to improving our therapeutical approach and lessening our Healthcare systems' burden.^[^ ^1^ ^]^

**Figure 1:**
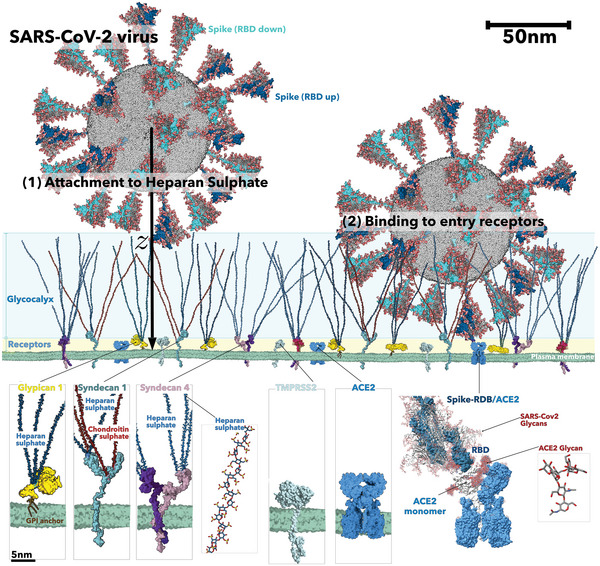
Representation of the SARS‐CoV‐2 virion landing on the membrane of a host cell in two steps: (1) Attachment to HS and (2) binding to the entry receptors. Spike glycoproteins are shown as a surface with all‐atom glycosylation and colored according to the receptor binding domain (RDB) conformation: up (dark blue, PDBid 6VXX) or down (cyan, PDBid 6VSB). On the host membrane, all‐atom structures of different components are represented as surface: syndecan 1 and 4, glypican, TMPRSS2, and the ACE2‐BT0 complex (PDBid 6M1D). The structure of the long heparan sulfate chain is highlighted as an inset and depicted as licorice. The binding between one ACE2 monomer and one spike glycoprotein RBD domain is highlighted as an inset with an all‐atom representation of a high‐mannose glycan. Multiple conformations (extracted from a molecular dynamics simulation of the ACERDB domain complex) of the ACE2 N‐glycans have been superimposed and depicted as licorice to highlight the N‐glycan trapping effect.

The viral life cycle starts with a game of multivalency when the viral particle starts the attachment process to the host cell (**Figure** **1**).^[^ ^2^ ^]^ Viruses recognize and bind to multiple cell surface receptors via protein/protein or protein/glycans interactions. Different viruses target different receptors with diverse sequences, structures, and cellular functions,^[^ ^3^ ^]^ with a strong preference for membrane proteins involved in cell adhesion.^[^ ^4^ ^]^ Nature tightly regulates the single viral protein/receptor interaction or affinity, and their combination into multivalent and multiplexed association profiles leads to a sharp response to gradients of receptors density and gives rise to the so‐called super‐selective binding.^[^ ^5^ ^]^ But the surface of most cells is coated with a complex mixture of carbohydrate chains expressed by proteoglycans and glycoproteins, forming the so‐called glycocalyx. Such a barrier can be as thick as tens of nanometers, creating steric protection that viruses must overcome before they reach the membrane and infect the cell (Figure 1). Many viruses,^[^ ^6^ ^]^ including influenza virus, herpes simplex virus, human immunodeficiency virus, and most coronaviruses, including SARS‐CoV‐2, have evolved the ability to exploit the cell surface proteoglycans^[^ ^7^ ^]^ as an attachment factor to aid their insertion.^[^ ^6a^ ^]^ The basic proteoglycan unit consists of a “core protein” with one or more covalently attached glycosaminoglycan (GAG) chains. In general, GAG‐binding domains on the membrane proteins of the virion envelope mediate initial attachment to proteoglycans. As shown in Figure 1, in the case of SARS‐CoV‐2, the virus spike glycoprotein (SPG) interacts with the negatively charged and linear GAG chains expressed by heparan sulfate (HS) proteoglycans.^[^ ^6b^ ^]^ There are two families of HS‐proteoglycans, the syndecans (from 1 to 4) and the glypicans (from 1 to 6). Figure 1 shows the structure of the most common Syndecan‐1 and ‐4 and Glypican‐1. The interactions between the SARS‐Cov‐2 SPG and the HS chains has been subjected to several studies, and it changes strongly with the HS sequence.^[^ ^6^, ^7^, ^8^ ^]^ Once the virus reaches the cell membrane, the specific binding of the receptor‐binding‐domain (RBD) of the SPG to the cellular‐entry receptors (R) takes place (Figure 1). Different entry receptors have been identified for coronaviruses, including human aminopeptidase N (APN; HCoV‐229E),^[^ ^6b^ ^]^ angiotensin‐converting enzyme 2 (ACE2; HCoV‐NL63, SARS‐CoV, and SARS‐CoV‐2)^[^ ^6^, ^9^ ^]^_ENREF_13, the transmembrane protease serine 2 precursor (TMPRSS2; SARS‐CoV‐2),^[^ ^9^ ^]^ scavenger receptor class B member 1 (SRB1, SARS‐CoV‐2),^[^ ^10^ ^]^ and dipeptidyl peptidase 4 (DPP4; MERS‐CoV).^[^ ^8c^ ^]^ The expression and tissue distribution of entry receptors influence viral tropism and pathogenicity, but the cell glycocalyx composition also plays a key role. In this work, we present a semi‐empirical model that predicts the ability of a virus to infect specific populations of cells in the host, viral tropism. The model is based on the description of the interactions between the virus and the host‐cell components, including an often neglected but key part of the cell: the cell glycocalyx. Here we described the different parts of the model with SARS‐CoV‐2 as the modeling subject. We predict its clinically observed host tropism and the differences observed with its predecessors, SARS‐CoV and MERS‐CoV.

## 2 A Simple Model for Viral Tropism

We can describe the host by its phenotype: the combination of receptors on its cell surface. Therefore, we can estimate tropism using a Langmuir‐like isotherm describing the cell surface coverage by virions depending on the cells’ phenotype. We compute two surface coverages, the glycocalyx surface coverage, *θ*_G_, and the receptor surface coverage, *θ*_R_. The glycocalyx surface coverage represents the fraction of the surface of the glycocalyx occupied by virions. The receptor surface coverage represents the fraction of the cell surface occupied by the virions successfully inserted into the glycocalyx.^[^ ^5^, ^11^ ^]^ *θ*_G_ is defined by the bulk viral titer (the number of virus copies per unit volume or viral load), the binding volume *v*_B_, and the grand canonical partition function in the glycocalyx surface *Q*_G_. While *θ*_R_ is defined by the viral titer in the glycocalyx, *ρ*_G_, the binding volume, *v*_B,_ and the grand canonical partition functions for the virus in the receptors’ surface *Q*_R_:

(1) $$\theta_{G}=\frac{\rho v_{B}Q_{G}}{1+\rho v_{B}Q_{G}}\wedge \theta_{R}=\frac{\rho_{G}v_{B}Q_{R}}{1+\rho_{G}v_{B}Q_{R}}$$

The surface coverage, *θ*_i_ (i = R, G), effectively normalizes the virus binding: *θ*_i_ = 0 indicates no interaction, and *θ*_i_ = 1 indicates full saturation of the available binding sites on the surface considered. Such an approach allows us to assess the nonlinear nature of viral binding and how it changes from zero to saturation for small changes in the cell's receptor/glycocalyx composition and the SGP affinities towards the GAGs present in the glycocalyx, viral size, spike number, and initial viral load. . The grand canonical partition functions, QG, and QR can be dissected into the product of attractive ($q_{GAG}^{\left(-\right)}$ and$q_{R}^{\left(-\right)}$) and repulsive ($q_{GAG}^{\left(+\right)}$) contributions.

(2) $$Q_{G}=q_{GAG}^{\left(-\right)}q_{GAG}^{\left(+\right)}\;and\;Q_{R}=q_{R}^{\left(-\right)}q_{GAG\;}^{\left(+\right)}$$

where the grand canonical partition functions are defined as:

(3) $$q_{GAG}^{\left(-\right)}=\sum_{j=1}^{N_{GAG}}\Omega_{GAG}\left(j\right)e^{-j\beta \varepsilon_{GAG}^{chain}}$$

(4) $$q_{R}^{\left(-\right)}=\sum_{j=1}^{N_{R}}\Omega_{R}\left(j\right)e^{-j\beta \left(\varepsilon_{R}+u_{R}\right)}\;$$

(5) $$q_{GAG}^{\left(+\right)}=e^{-\beta u_{GAG}}$$

where *N*_GAG_ and *N*_R_ are the number of GAG chains and receptors per binding unit, respectively. Ω_*GAG*_(*j*) and Ω_*R*_(*j*) are the number of possible combinations between the viral SGP and the GAG chains and the receptors, respectively. $\varepsilon_{GAG}^{chain}$ is the binding energy of the viral SGP and the single GAG chain and *ε*_*R*_ is the binding energy of the viral SGP and the single‐entry receptor. *u_R_* is the receptors’ steric potential arising from the compression of the receptor glycans trapped during the SPG binding. As shown in Figure 1, lower right panel, the ACE2 receptor is glycosylated; therefore, when the RDB domain of the SGP (also glycosylated) binds to the ACE2 receptor, one or more of the glycans on the surface of the ACE2 receptor can be trapped in the binding site. TMPRSS2 is not glycosylated; therefore, *u_R_* =0 for TMPRSS2. *u_GAG_* is the steric potential arising from the insertion of the virus into the glycocalyx or GAG chains and is the inverse thermodynamic temperature. Equations (1)–(5) allow us to estimate the virus tropism as a function of the virion interaction with tcell glycocalyx and the entry receptors. Each of these interactions can be derived separately and ultimately depends on the experimentally measured equilibrium binding affinities of the virus spike glycoprotein towards the GAGs and entry receptors. Further derivation details are provided in the Supporting Information.

### 2.1 The Energetics of the Virion Insertion into the Cell Glycocalyx

**Figure 2:**
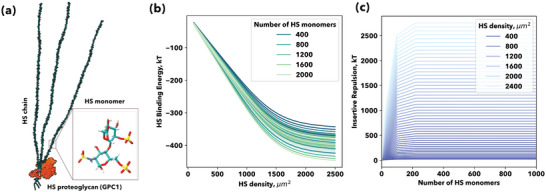
The insertion steps into the glycocalyx. a) All‐atom representation of the glypican‐1 proteoglycan together with an inset of an HS highly sulfated, IdoA(2S)‐GlcNS(6S), monomer. b) Heparan sulfate (HS)‐spike protein binding: collective binding interaction (kT) behavior with an increasing number of monomers per HS chain and HS chains density per µm^2^. **c)** Heparan sulfate (HS)‐spike protein insertion repulsion: steric repulsion evolution with an increasing number of HS monomers, chain and HS chains density per µm^2^.

As depicted in Figure 1(1), during the insertion process into the glycocalyx, coronaviruses bind to the HS chains attached to proteoglycans. Each GAG chain consists of a variably sulphaated repeating disaccharide unit, as depicted for HS in Figures 1 and **2**a. Only a fraction of these GAG units bind to the virion SPG, in the case of HS 8 to 10 monomers or binding motifs.^[^ ^12^ ^]^ Therefore, the total binding energy between a single GAG chain and the virion, $\varepsilon_{GAG}^{chain}$ is proportional to the number of binding motifs, *N_L_* per GAG chain (where *N_L_* is a fraction of the number of monomers per chain). The more binding motifs or number of monomers, the higher the attractive energy (Figure 2b). This energy also increases with the number of GAG chains, which is proportional to the density of proteoglycans (Figure 2b). The repulsive energy generated by the insertion of the virus into the GAGs, $q_{GAG}^{\left(+\right)}$, increases dramatically with the number of binding motifs per chain and the number of chains or proteoglycan density (Figure 2c).

### 2.2 The Energetics of the Cell Receptor‐Specific Binding. Glycans as a Source of Steric Repulsion

**Figure 3:**
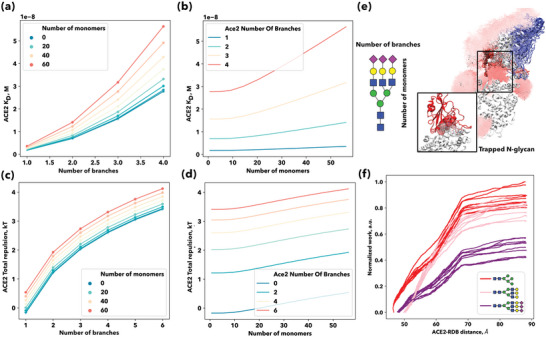
The effect of glycosylation in recognizing and binding the spike protein to the host cell entry receptor. a,b) ACE2‐RBD Kd variation with glycan morphology: ACE2‐RBD dissociation constant plotted as a function of the number of branches and colored according to the number of monomers per branch in (c) and vice versa in (d). c,d) ACE2 Glycans steric potential variation with glycan morphology: the steric potential generated by glycan compression in kT units is plotted as a function of the number of branches and colored according to the number of monomers per brand in (e) and vice versa in (f). e) ACE2‐RBD complex dissociation: multiple snapshots from one of the out‐of‐equilibrium simulations of the dissociation of the ACE2‐RBD complex are superimposed for the glycans and RBD domain. The RBD domain is colored according to the distance to the binding site (red bound, blue unbound for the high‐mannose system. Glycans are represented as lines and colored radially in shades of pink. An all‐atom representation of the glycan trapped in the binding site is provided as an inset. f) Collection of 10 normalized work profiles along the reaction coordinate for three different N‐glycans: high mannose type (red), complex type (pink), and complex type with terminal sialic acid (purple).

Once the virion is inserted into the glycocalyx, it can bind to the ACE2 receptor via the RBD of the SGP (Figure 1(2)). This interaction is not purely attractive due to the compression of the glycans at the binding site (Figures 1 and **3**e. Glycosylation is a common process in the human body, in which a chain of sugars, or glycan, is attached to a protein. The composition of these glycans can vary widely between species, with humans' glycans being particularly large and complex.^[^ ^13^ ^]^ Both host cell and viral proteins are glycosylated, and the post‐translational glycosylation process plays a major role in infection virulence and interaction with the host immune system.^[^ ^14^ ^]^ The SGP of SARS‐CoV‐2 typically contains 22 N‐glycans ^[^ ^15^ ^]^ and 2 O‐glycans.^[^ ^15a^ ^]^ The SGP glycans shield the virus from the immune system,^[^ ^16^ ^]^ but it also seems to regulate the opening and closing of the RBD domain.^[^ ^17^ ^]^

In Figure 3, we analyzed the influence of the trapped glycans on the dissociation constant ($K_{D}=1/K_{A}=\rho^{0}e^{E_{R}}$, where $\rho^{0}=1\;M$ and *E_R_* = −*ln*) of the ACE2‐spike protein complex. The number of branches or antennas and the branch length (number of monomers) increases the K_D_ of the complex (Figure 3a). Therefore, the same SGP interacting with the same receptors can have different *K*_D_ values due to differences in glycosylation. Experimental studies performed in different human cells (hence different glycosylation) report different dissociation constants, proteins expressed in HEK293T reported a *K*_D_ = 1.2, 5.09 nM,^[^ ^18^ ^]^ FreeStyle293F *K*_D_ = 34.6 nM,^[^ ^19^ ^]^ while proteins expressed in insect Sf9 cell, reported *K*_D_ = 4 nM.^[^ ^20^ ^]^ As shown in Figure 3b, for a glycan with the same number of branches, the number of monomers, and the length of the branches, also increase the *K*_D_ of the complex, decreasing the binding affinity. By chemically modifying various glycans' positions and measuring binding affinity using surface plasmon resonance (SPR), Allen et al.^[^ ^21^ ^]^ showed the influence of ACE2 glycosylation on the binding of the RBD domain of the SARS‐CoV‐2 spike protein experimentally. First of all, they found that deglycosylated ACE2 is capable of binding the SARS‐CoV‐2 SGP. The predominant type of glycan observed across all five WT‐ACE2 glycosylated sites were complex‐type glycans with two or three branches. When ACE2 was expressed with a solubilized variant of *β*‐galactoside *α*‐2,6‐sialyltransferase I (ST6), increasing the complexity of the glycans expressed in ACE2, the *K*_D_ of the complex increased from 58.7 nM (WT) to 98.6 nM, therefore decreasing the binding affinity of the complex. ACE2 treated with sialidase, glycans with fewer monomers have a higher affinity for SARS‐CoV‐2 than WT ACE2, *K*_D_ 30% lower. Chemical modifications not affecting the glycan morphology (number of branches or number of monomers per branch) did not impact ACE2 binding to a significant extent. But when all N‐linked glycans on ACE2 were converted to oligomannose‐type, less complex glycans, the *K*_D_ increased by ≈50%, corresponding to a decrease in binding affinity. These differences though subtle, are in perfect agreement with our model. In Figure 3c,d, we show that the increasing glycan complexity (branching and monomer length) increases the total ACE2‐RBD repulsion potential due to the steric potential generated by the glycan compression in the binding site, decreasing binding affinity and increasing *K*_D_ (Figure 3a,b). The difference between an insect glycan (Figure 3a) and a more complex glycan (human‐like) with 3 to 4 branches is nearly 20 nM (≈17.7 kT for one glycan trapped in the binding region). According to our model, differences in *K*_D_ can be as high as 50 nM (≈16.8 kT) between a single‐branched short glycan and multiple‐branched complex human long glycan, like the ones found in the human lungs (Figure 3b).^[^ ^22^ ^]^ To understand at the microscopic level the role of the trapped glycans in the RDB‐ACE2 binding interface, we performed several steered molecular dynamics (SMD) simulations of the dissociation of the complex for two different glycosylation patterns in the ACE2 receptor. We simulated three systems: high mannose type (DManpa1‐6[DManpa1‐3]DManpa1‐6[DManpa1‐3]), complex type (DGalpb1‐4DGlcpNAcb1‐2DManpa1‐6[DGalpb1‐4DGlcpNAcb1‐4[DGalpb1‐4DGlcpNAcb1‐2]DManpa1‐3]DManpb1‐4DGlcpNAcb1‐4DGlcpNAca1), and a complex type glycan with sialic acid terminal (DNeup5Aca2‐3DGalpb1‐3DGlcpNAcb1‐6[DNeup5Aca2‐3DGalpb1‐4DGlcpNAcb1‐2]DManpa1‐6[DNeup5Aca2‐3DGalpb1‐3DGlcpNAcb1‐2DManpa1‐3]DManpb1‐4DGlcpNAcb1‐4DGlcpNAcb1) (Figure 3e, left to right). After equilibrating the complex with the desired glycosylation pattern, we computed ten independent SMD pulling trajectories for each system by adding a harmonic restraint on the distance between the center of mass of the two proteins. In Figure 3f, we depicted the obtained normalized work profiles for the three systems. The work required to break the system with a longer and more complex glycan (the complex type with sialic acid terminal), Figure 3f) is lower than the one required to break the complex type system which is also lower than the work required to break the high‐mannose system. Hence, the binding affinity of the complex decreases with the number of monomers of the glycans, and its *K*_D_ increases. This effect is due to the conformational volume reduction (entropic contribution) of the trapped glycan in the complex interface.

## 3 Viral Tropism in a Nutshell

**Figure 4:**
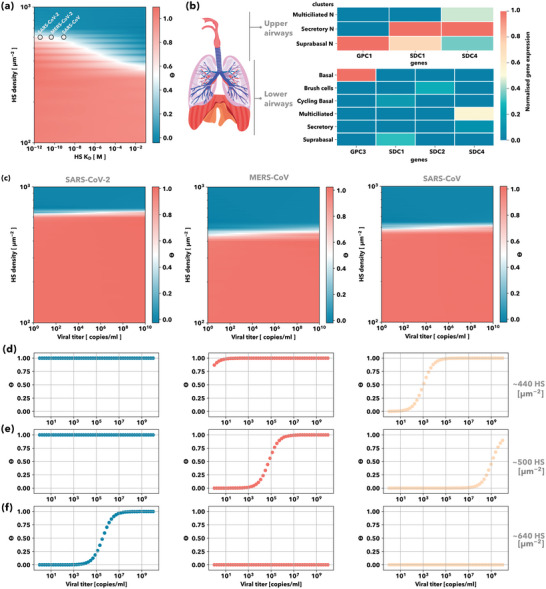
Viral tropism: cell proteoglycans density and membrane receptors synergy. a) Cell Surface coverage by viral particles variation with HS density and HS‐spike binding affinity: Contour plot of the cell surface coverage by the viral particles as a function of the HS density and the spike protein‐HS binding affinity. Surface coverage equal to 1 implies that the initial viral titer successfully attaches to the cell surface. SARS‐CoV‐2, SARS‐CoV, and MERS‐CoV spike proteins HS binding affinities are displayed. b) Proteoglycans gene expression across cell types for the upper and lower human airways: Each gene is normalized to its maximum value across all tissues. The tissues correspond to the indicated positions along the airway from the nasal to the distal lung. Dataset obtained from Durant et al. Initial viral titer threshold for binding. c) Cell surface coverage by viral particles variation with HS density and initial viral titer: Contour plot of the cell surface coverage by the viral particles as a function of the HS density and the initial viral titer for SARS‐CoV‐2 (left), MERS‐CoV (middle), and SARS‐CoV (right). Cell surface coverage by viral particles variation with the initial viral titer for d) ≈440, e) ≈500, and f) ≈640 HS chains per µm^2^; for SARS‐CoV‐2 (left), MERS‐CoV (middle), and SARS‐CoV (right).

The attractive/repulsive interactions described above define the viral tropism via the grand canonical partition function, *Q*_*G*/*R*_. In our model, different cell phenotypes are characterized by the expression of proteoglycans (proteoglycans density, µm^2^) and entry receptors (receptors density, µm^2^). Likewise, viruses can be described by the binding affinity of their SGP to the GAGs and entry receptors. In **Figure** **4**a, we can observe how the GAG density and the virus spike protein‐GAG KD affect *θ*_G_ for cells with a specific proteoglycan density. Above a certain proteoglycan density, the virus‐cell interaction is switched off, known as range selectivity.^[^ ^11a^ ^]^ This change from bound to unbound occurs within a narrow range of GAG densities. We can use the experimental data collected for SARS‐CoV‐2, SARS‐CoV, and MERS‐CoV binding to Heparin^[^ ^8b^ ^]^ to show that SARS‐CoV‐2 has a higher affinity towards HS: this allows SARS‐CoV‐2 to bind to higher HS densities regions, compared to the other coronavirus (Figure 4a). By analyzing the HS‐proteoglycan expression on the different tissues and cells found in the human upper and lower airways, Figure 4b, we found that the upper airways have a proteoglycan density which favors the attachment of SARS‐CoV‐2, but not SARS‐CoV or MERS‐CoV. The ideal location for the attachment of SARS‐CoV and MERS‐CoV is in regions with lower proteoglycan densities, such as the lower airways.

Conversely, SARS‐CoV‐2 can also attach and replicate in the upper airways' epithelium, thus infecting both the upper and the lower respiratory tract and navigating the airways more efficiently, as reported by virological assessment of SARS‐CoV‐2, SARS‐CoV and MERS‐CoV patients.^[^ ^23^ ^]^ Moreover, we can observe in Figure 4c that the affinity of the virus for the GAG (HS)s determines the initial viral titer required for successful attachment. At low GAG (HS) densities, Figure 4d, very low initial viral titers result in full cell surface coverage for SARS‐CoV‐2 (Figure 4d, blue) and MERS‐CoV (Figure 4d, middle), while SARS‐CoV needs at least 10^3^ copies ml^−1^ in bulk to have 50% of cell surface coverage. At medium GAG (HS) densities, Figure 4e, SARS‐CoV needs at least 10^9^ copies ml^−1^ to achieve 50% of surface coverage (Figure 4e, beige) and MERS‐CoV requires 10^5^ copies ml^−1^ (Figure 4e, orange‐red). At high GAG (HS) densities, Figure 4f, MERS‐CoV and SARS‐CoV cannot bind under any condition, while SARS‐CoV‐2 binds for initial viral titers above 10^3^ copies ml^−1^ (Figure 4f, blue). Respiratory samples extracted from SARS‐CoV‐2 patients with mild and severe COVID‐19 symptoms revealed viral titers between 10^3^ and 10^9^ copies ml^−1^.^[^ ^24^ ^]^

**Figure 5:**
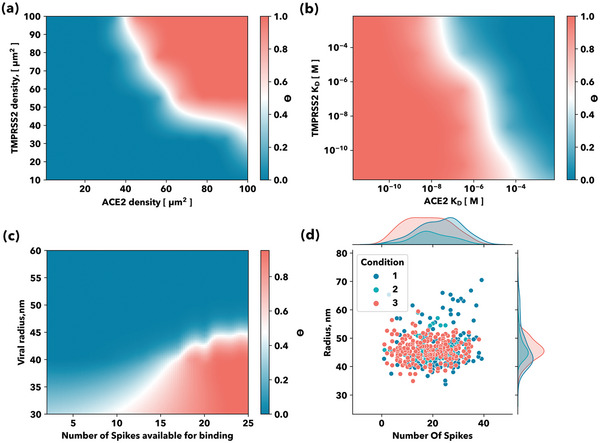
Viral tropism: Entry receptors binding synergy: a) Contour plots of the cell surface coverage by the viral particle as a function of the ACE2 and TMPRSS2 cell densities and b) affinity towards the viral spike protein. c) Viral morphology: radius and spike‐proteins available for binding influence on viral the number of viral particles attached to the cell surface: Contour plot of the virus titer on the cell surface (log) as a function of the virion radius and the number of spikes proteins in open conformation available for binding. d) Viral morphology: radius and number of protein spikes. Probability distributions of the different SARS‐CoV‐2 viral radius and number of protein spikes.^[^ ^30^ ^]^

Several receptors have been found responsible for SARS‐CoV‐2 entry, and we can expand our analysis to all of them. But, for the sake of simplicity, we focus on the most widely discussed elements in the SARS‐CoV‐2 infection, the receptors ACE2 and TMPRSS2.^[^ ^25^ ^]^ **Figure** **5**a shows how successful viral attachment happens when the receptors act synergistically. Cells for which the number of ACE2 receptors accounts for less than 40% of the total receptor density per µm^2^, as the olfactory sensory neurons (OSN),^[^ ^26^ ^]^ have low cell surface coverage (low viral titer on the surface) for any percentage of TMPRSS2. While for cells expressing high densities of ACE2, > 60%, even moderate values of TMPRSS2 are enough to obtain high cell‐surface coverage values. Many cells from the human airways,^[^ ^26^, ^27^ ^]^ including olfactory epithelial sustentacular cells, appertain to this case, which explains why SARS‐CoV‐2 infection of non‐neuronal cell types leads to olfactory dysfunction (anosmia) in patients with COVID‐19.

Mutations of the RBD domain of the SARS‐CoV‐2 SGP are worrying due to the higher transmissibility of these variants, especially the Gamma, Beta, Alpha, Delta, and Omicron,^[^ ^28^ ^]^ and their ability to hide the virus from antibodies^[^ ^28^, ^29^ ^]^ and cause reinfection.^[^ ^28e^ ^]^ The N501Y mutation shared by all the Gamma, Beta, and Alpha variants increases the binding affinity of the RBD domain towards the ACE2, decreasing its *K*_D_.^[^ ^28d^ ^]^ However, for an optimum ratio of receptors (ACE2, TMPRSS2) densities, our model does not predict significant changes in cell surface coverage with decreasing ACE2‐RBD‐domain *K*_D_ (Figure 5b). For high ACE2 *K*_D_ values (low binding affinity), on the other hand, changes in the affinity towards TMPRSS2 might lead to a decrease in viral titer on the cell surface, according to our model (Figure 5b).

Finally, we found that both the virion morphology and the number of available RBD domains in open conformation available for binding to the ACE2 affect virus titer on the cell surface. Smaller virions ≈40 nm radius are successful in attaching to the host cell with less number of spikes proteins available for binding to the entry receptors, while a bigger virus with the same number of spikes available for binding would be less successful in this case and the virus titer on the cell surface will be lower Figure 5c. Cryogenic electron microscopy on SARS‐CoV‐2 virions populations has revealed an average radius of ≈45 nm with a standard deviation of ≈10 nm, with individual virions containing 24 ± 9 spike proteins not uniformly distributed and highly flexible.^[^ ^30^ ^]^ Interestingly, as shown in Figure 5c, there is a very nonlinear response with the morphology of the virus, and indeed viruses with a radius bigger than 45 nm are less effective at attaching to cells. According to our model for bigger virions to be as effective as *r* = 45 nm, they should have a bigger number of spike proteins on their surface. By fitting the measurements provided by Ke et al,^[^ ^30b^ ^]^ Figure 5d, we observe that for all three preparations there is a clear distribution of size but the number of protein spikes per virion distributions is very broad, indicating that the size of the virus is the critical parameter for viral attachment.

## 4 Conclusion

Overall, our model can explain the viral tropism of SARS‐CoV‐2 in terms of interactions with the different host cell components. We find that viruses are extremely selective, not only in terms of entry receptors distribution on the host cell but also on the spike protein affinity towards other key elements of the cell phenotype. The glycocalyx of the cell plays a major role in viral infection. We showed how the repulsion potential generated by the long heparan sulfate chains can dramatically the number of virions that can bind to the entry receptors, hence increasing the initial viral load required for infection. We have also shown that the role of the entry receptors’ glycosylation is not enthalpic but entropic, decreasing the binding affinity of the RBD‐ACE2 complex at increasing morphological complexity. Finally, we have shown how the size of the viral particle is a critical parameter with a nonlinear response, and therefore, induces a selection of the optimal viral size for infection.

## 5 Experimental Section

### Out‐of‐Equilibrium Molecular Dynamics

The ACE2‐RDB SARS‐CoV2 complex was extracted from the Cryo‐EM PDB structure 6M17.^[^ ^31^ ^]^ The protein complex presented no mutations or structural gaps. Three different systems (different glycan types) were generated by adding glycans using the Glycam web server Glycoprotein Builder tool,^[^ ^32^ ^]^ the high mannose type (DManpa1‐6[DManpa1‐3]DManpa1‐6[DManpa1‐3]), the complex type (DGalpb1‐4DGlcpNAcb1‐2DManpa1‐6[DGalpb1‐4DGlcpNAcb1‐4[DGalpb1‐4DGlcpNAcb1‐2]DManpa1‐3]DManpb1‐4DGlcpNAcb1‐4DGlcpNAca1), and the complex type glycan with sialic acid terminal (DNeup5Aca2‐3DGalpb1‐3DGlcpNAcb1‐6[DNeup5Aca2‐3DGalpb1‐4DGlcpNAcb1‐2]DManpa1‐6[DNeup5Aca2‐3DGalpb1‐3DGlcpNAcb1‐2DManpa1‐3]DManpb1‐4DGlcpNAcb1‐4DGlcpNAcb1). The three systems were protonated at pH 7.4 and solvated (150 mM NaCl) using a TIP3P^[^ ^33^ ^]^ water model and Charmm36m^[^ ^34^ ^]^ protein, glycans, and ions force field. The final simulated systems consisted of 240 K atoms, resulting in a cubic box simulation cell of 12 ×12 ×17 nm. All simulations were performed with Gromacs v2019.2^[^ ^35^ ^]^ with a 2fs integration step. 2000 steps of minimization (steepest descent algorithm) were performed, followed by 5000 steps of isotropic NPT at 300 K, using the velocity‐rescaling thermostat^[^ ^36^ ^]^ and Parrinello–Rahman barostat.^[^ ^37^ ^]^ After 50 000 steps of NVT, the system was fully equilibrated and ready for production, which was performed in the NVT ensemble for 5 ns. Ten steered molecular dynamics simulations were performed for each system using PLUMED2.6.^[^ ^38^ ^]^

### Synthetic Data Generation

In **Table** **1**, the model parameters fixed to generate the data for each of the figures displayed in this article are specified.

**Table 1:** Model fixed parameters used to generate the synthetic data

	Figure 2	Figure 3	Figure 4a	Figure 4c	Figure 4d	Figure 4e	Figure 4f	Figure 5a	Figure 5b	Figure 5c
Viral radius [nm]	50	50	50	50	50	50	50	50	50	
Viral titer [copies ml^−1^]	3.01E+07	6.02E+07	3.01E+07	3.01E+07	3.01E+07	3.01E+07	3.01E+07	6.02E+07	6.02E+07	6.02E+07
Number of Spikes	15	15	15	15	15	15	15	15	15	
Spike length [m]	1.45E‐08	1.45E‐08	1.45E‐08	1.45E‐08	1.45E‐08	1.45E‐08	1.45E‐08	1.45E‐08	1.45E‐08	1.45E‐08
HS‐Spike binding energy [kT]	−16			−25,−18,−16	−25,−18,−16	−25,−18,−16	−25,−18,−16	−23	−23	−23
HS fraction of binding motif	0.1		0.1	0.1	0.1	0.1	0.1	0.1	0.1	0.1
HS chain length [m]			1.3E‐07	1.3E‐07	1.3E‐07	1.3E‐07	1.3E‐07			1.5E‐07
HS number of chains, per µm^2^					440	500	640			100
HS Kuhn length [m]	9E‐09		9E‐09	9E‐09	9E‐09	9E‐09	9E‐09			9E‐09
ACE2 mean binding distance		2.7E‐09						2.7E‐09	2.7E‐09	1E‐09
ACE2 glycan branch length								1.25E‐09	1.25E‐09	1.5E‐09
ACE2 glycan number of branches								2	2	3
ACE2 number of trapped glycans		2						2	2	2
ACE2‐spike *K*_D_		2.06E‐09						2.06E‐09		2.06E‐09
Number of ACE2 per µm^2^		56							56	45
Number of ACE2 binding sites		3						3	3	3
Number of TMPRSS2 per µm^2^		100							100	100
TMPRSS2‐spike K_D_		2.06E‐09						2.06E‐09		1.52E‐08
Number of TMPRSS2 binding sites		1						1	1	1
Cell size [m^2^]	1E‐14	1E‐14	1E‐14					1E‐14	1E‐14	1E‐14

## Conflict of Interest

The authors declare no conflict of interest.

## Author Contributions

J.B. and G.B. performed the mathematical derivation of the model. J.B. coded the model. S.A.G. performed the all‐atom simulations and the generation, analysis, and representation of the synthetic data presented in the manuscript. All three authors wrote the manuscript.

## Supporting information

Supporting Information

## Data Availability

The source code to reproduce the data presented in this work is publicly available in https://dev.azure.com/MolecularBionics/SARS%20Binding/_git/Source.
