# Correction to “The Role of Host Cell Glycans on Virus Infectivity: The SARS‐CoV‐2 Case”

**DOI:** 10.1002/advs.202406342

**Published:** 2024-07-03

S. Acosta‐Gutiérrez, J. Buckley, G. Battaglia, The Role of Host Cell Glycans on Virus Infectivity: The SARS‐CoV‐2 Case. Adv. Sci. 2022, 10, 2201853. https://doi.org/10.1002/advs.202201853

The Acknowledgement section should include: “SAG acknowledges support from an AGAUR Beatriu de Pinós MSCA‐COFUND Fellowship (project 2020‐BP‐00177).”

The section should be modified to: “The authors thank the ERC for consolidator award (CheSSTaG 769798) for sponsoring this work and JB studentship, the Children with Cancer UK for the research project (16‐227), and EPSRC/SomaNautix Healthcare Partnership EP/R024723/1 for sponsoring part of SAG salary. SAG acknowledges support from an AGAUR Beatriu de Pinós MSCA‐COFUND Fellowship (project 2020‐BP‐00177). G.B. thanks the EPSRC Established Career Fellowship (EP/N026322/1) for sponsoring part of his salary. The authors thank Dr. Fran Crawford, Dr. Denis Cecchin, Dr. Loris Rizzello, Dr. Laura Rodriguez‐Arco, Dr. Azzurra Apriceno, Dr. Claudia Di Guglielmo, Dr. Joseph Forth, Dr. Yangwei Deng, Dr. Diana Matias, Dr. Diana Leite, Dr. Cristina Picken, Dr. Virginia Gouveia, Mr. Valentino Barbieri, Ms. Chiara Cursi, Ms. Barbara Ibarzo Yus, and Mr. Miko Sipin all part of the Molecular Bionics COVID Taskforce for the very insightful discussions. The authors also thank Dr. Stefano Angioletti‐Uberti for their useful feedback.”

The following affiliation should be displayed for all three authors and not the last one only:

“Institute for Bioengineering of Catalunya (IBEC), The Barcelona Institute of Science and Technology, Barcelona, Barcelona, 08028 Spain”

