# Supplementary material for: The Role of Host Cell Glycans on Virus Infectivity: The SARS‐CoV‐2 Case
Source: Adv Sci (Weinh). 2022 Nov 23;10(1):2201853. doi: 10.1002/advs.202201853 (PMC9811451; doi:10.1002/advs.202201853)
Supplement: Supplementary file 1 — Supporting Information [file ADVS-10-2201853-s001.pdf]

The role of host cell glycans on virus infectivity: The SARS-CoV-2 case – Supplementary Information

Silvia Acosta-Gutiérrez, Joseph Buckley and Giuseppe Battaglia

September 4, 2022

Theoretical Model

As discussed in the main text, binding between coronaviruses and the host cells proceeds in a two step process. First, the virus binds to the glycosaminoglycans (Heperan Sulfate), the long-chain sugars that coat the cell surface. After binding to the glycosaminoglycans, the virus then can form bonds with the entry-receptors on the surface, particularly ACE2 and TMPRSS2 in the case of SARS-CoV-2.

Following the derivation of a simple analytical model for the adsorption of multivalent particles on a surface [1], we will define the glycocalyx surface coverage,θ_G, as the probability that a virus will be bound to a GAG chain at a given site, and the cell-surface coverage, θ_R, as the probability that a virus will be bound to a receptor at a given site. As pointed out in the original work of Veracochea et.al. [1] the predicted Langmuir dependence of the surface covarge (θ) on bulk concentration of the multivalent guest particles has been observed experimentally for functionalized colloids [2], nanoparticle-based drug delivery systems [3, 4, 5, 6] and multivalent host-guest interaction interfaces [7]. In equation 1 in the main text, we give the general form of the surface coverage, which we will specialise to deal with each case, giving

$$\theta_G = \frac{\rho v_B Q_G}{1 + \rho v_B Q_G} \qquad \text{and} \qquad \theta_R = \frac{\rho_G v_B Q_R}{1 + \rho_G v_B Q_R}, \tag{1}$$

where the binding volume, v_B, is determined from the viral radius, R, and the spike length, d, as $v_B = \pi((R+d)^3 - \frac{\pi}{3}R^3)$ and ρ is the viral bulk concentration, and ρ_G is the viral concentration in the glycocalyx. The cell-surface coverage can be linked to the glycocalyx surface coverage as $\rho_G = \frac{\theta_G}{v_B}$, however, in order to limit the complexity for our calculation, we treat ρ_G as a parameter, effectively decoupling the system.

The partition functions, Q_G and Q_R, contains information about the possible states of the system when the virus is bound to either the surface or the GAG chains respectively. We can calculate the partition function taking the product of the individual contributions to the respective partition function.

For Q_G, we have contributions from the GAG binding, $q^{(-)}_{GAG}$ as well as a contribution from the steric repulsion arising from insertion into the chain, $q^{(+)}_{GAG}$,

$$Q_G = q^{(-)}_{GAG} q^{(+)}_{GAG}. \tag{2}$$

Likewise, for Q_R we have contributions from the receptor binding $q^{(-)}_R$, as well as the steric repulsion generated by the surrounding GAGs $q^{(+)}_{GAG}$,

$$Q_R = q^{(-)}_R q^{(+)}_{GAG}. \tag{3}$$

It should be noted that $q^{(+)}_{GAG}$ has slightly different form in each equation, due to differences in the geometry of binding in the different cases. The individual partition functions will be discussed in more detail in the following subsections.

GAG Binding

Each GAG chain contains multiple sites to which the spike can bind. The number of these binding sites, N_L is some fraction of the number of units, N_p. The number of units can be calculated from the average GAG chain length, d_{GAG} and the GAG Kuhn length, b_{GAG} as $N_p = (d_{GAG}/b_{GAG})^2$.

Only a single bond can form between a given GAG chain and the virus. This is likely due to the mechanical properties of the GAG chain, suggesting the entropic cost of forming an additional bond is too high. We can, therefore, state the partition function for a single GAG chain binding to the virus in terms of the number of spikes available for binding, N_S, and the GAG-spike binding energy in solution ϵ^{motif}_{GAG} [8]:

$$q^{chain}_{GAG} = N_L N_S \exp\left(-\frac{\epsilon^{motif}_{GAG}}{k_B T}\right). \tag{4}$$

Then, we can calculate the total partition function for GAG-spike binding from the binding energy of a given chain, $\epsilon^{chain}_{GAG} = -\ln(q^{chain}_{GAG})$, and the number of chains, as [1]

$$q^{(-)}_{GAG} = \sum_{i=0}^{N_{GAG}} \binom{N_S}{i} \binom{N_{GAG}}{i} i! \exp\left(\frac{-i\epsilon^{chain}_{GAG}}{k_B T}\right). \tag{5}$$

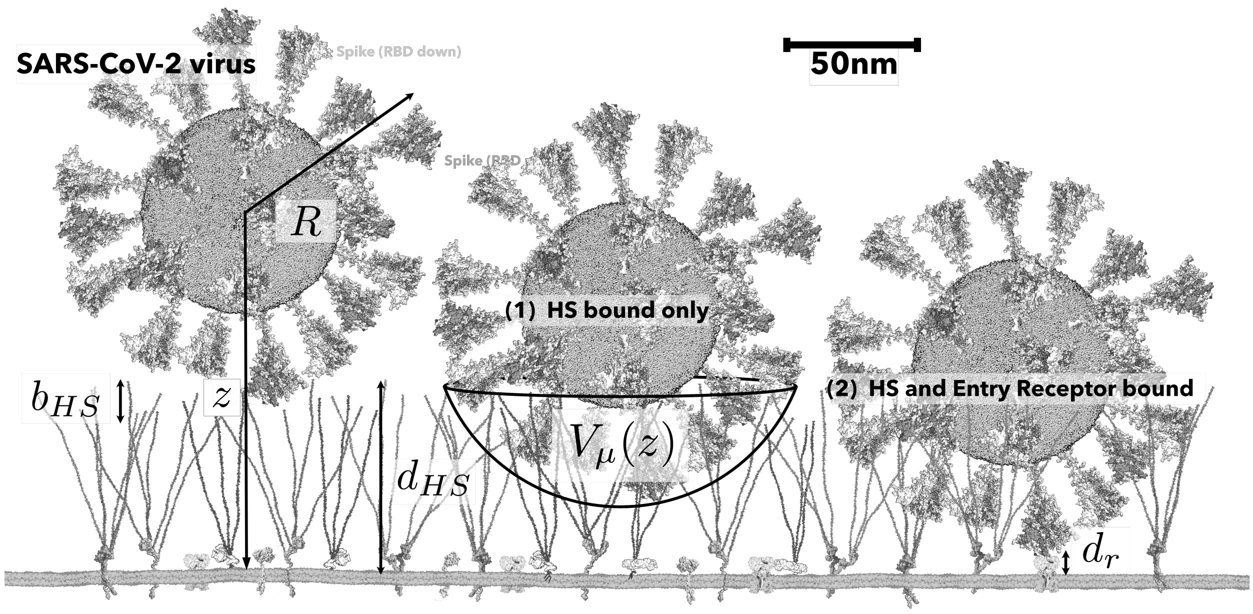

Figure S1: Virion landing onto the host cell membrane. Description of the geometrical parameters of the model.

GAG Repulsion

As multiple GAG chains will bind to a single proteoglycan, we expect the density of the HS chains to be non-uniformly distributed, with areas of locally high density around each proteoglycan. This means when the virus is bound only to GAG chains, there will be effectively a high local density then when the virus is bound to the receptors.

We, therefore, have to consider two cases for the steric repulsion. First, when the virus is bound only to GAG chains, and the second when the virus is bound to the receptors. When the virus is bound to receptors, the steric repulsion depends on the global GAG density, ρ_{HS}, i.e. the average density over the entire cell. However, when the virus is bound only to GAG chains, we use the local GAG density to account for the clustering together of GAG chains.

We can then calculate the energy of insertion in terms of the density of chains, the inserted volume, $V_\mu(z)$, and the insertion parameter, δ [4]. This gives

$$u_{GAG} = k_B T V_\mu(z) \rho_{GAG} \left(1 - \delta_p^2\right)^{\frac{9}{4}}. \tag{6}$$

From this expression, we can calculate the partition function as

$$q_{GAG}^{(+)} = \exp\left(-\frac{u_{GAG}}{k_B T}\right) \tag{7}$$

The inserted volume can be calculated geometrically (Figure S.Fig.S1) by considering the distance from the surface, z, as

$$V_\mu(z) = \begin{cases} \frac{1}{3}\pi z^2(3R - z), & \text{for } z < 2R \\ \frac{4}{3}\pi R^3, & \text{for } z \geq 2R. \end{cases} \tag{8}$$

Insertion Parameter

The insertion parameter quantifies how far into the brush the virus has penetrated, and is given as the ratio between the brush length and the distance between the virus and surface. It can be expressed in terms of the GAG chain length, d_{GAG}, the distance between the virus and the cell surface, z, and the receptor tether length, d_r, as

$$\delta_p = \frac{z}{d_r + d_{GAG}}. \tag{9}$$

In the presence of receptor-spike binding, the cell-virus distance will be equal to the receptor tether length, d_r, Fig.??. However, in the absence of receptor-spike binding, situation is more complex.

We will number the binding sites of a GAG chain, from $n = 1, ..., N_L$, where $n = 1$ is the site farthest from the cell surface. We can then define the distance between two sites, n and $n + 1$, as d_n. It is not possible to identify each distance between sites, so we will assume that the distances can be approximated by the average distance, an assumption that holds for long chain lengths with roughly equal distribution of sites. We can then express the inter-site distance as

The virus-cell distance can then be expressed as a function of the closest binding site to the surface, n;

$$z = \begin{cases} d_r & \text{for receptor-spike binding} \\ d_r + d_{GAG} - n\frac{b_{GAG}\sqrt{N_P}}{(N_L+1)} & \text{otherwise.} \end{cases} \tag{10}$$

Mean Field Approximation

As we cannot know the distance of each virus from the cell surface, we will apply a mean field approximation, treating the repulsion as the repulsion at the average distance.

To do this, we must find the probability that a given site, n, is the closest bound site to the surface on a given chain. This can be expressed in terms of the probability that no bonds are formed on any GAG chain at a given site, $p_\kappa = (1-\kappa)^{N_{GAG}}$, where κ is the probability a given site is bound (which can be calculated from the binding energy of a given site as described in equation (16))

$$p^L(n) = (1-p_\kappa)p_\kappa^{N_L-n}. \tag{11}$$

The expectation value of the cell-virus distance is then given by

$$\langle z \rangle = d_r + \sum_{n=1}^{N_L} p_L(n) \left(d_{GAG} - n\frac{b_{GAG}\sqrt{N_P}}{(N_L+1)} \right). \tag{12}$$

Receptor Interaction

As there are multiple possible receptors that the spike can bind to, we need to take a different but equivalent approach for calculating the receptor-spike partition function. First, we define the partition function as [9]

$$q_R^{(-)} = \exp\left(-\frac{\epsilon_R + u_R}{k_B T}\right) - 1. \tag{13}$$

where ϵ_R is the binding energy of the single receptor-spike RDB domain and u_R is the steric repulsion arising from the receptor glycans trapped in the binding site, Fig. S2. Then, we can define the energy of binding in terms of the probability that a given site is unbound, p_i.

$$\frac{\epsilon_R + u_R}{k_B T} = \sum_i \ln p_i + \frac{1}{2}\left(1 - p_i\right), \tag{14}$$

The probability of a receptor and the spike RBD domain being unbound can be calculated by closure (i.e. all probabilities must sum to 1)[10]. Defining p_{ij} as the probability of receptor i is bound to the RDB domain j, we have

$$p_i + \sum_j p_{ij} = 1. \tag{15}$$

Finally, we can calculate p_{ij} can be defined as the product of the probability that both i and j are unbound and the probability that a bond will form, which is given by the Boltzmann factor – the negative exponential of the binding energy. This gives the expression

$$p_{ij} = p_i p_j exp\left(\frac{\epsilon_R + u_R}{k_B T}\right). \tag{16}$$

A full derivation for this can be found in the original work by Varilly et al. [10].

Trapped receptor N-glycans generated steric repulsion

The binding energy between the spike and a given receptor has both an attractive component, arising from the binding interaction, and a repulsive component, arising from the steric repulsion caused by trapped glycans, as discussed in the main body.

TMPRSS2 is not gycosylated and has only one binding site, therefore $u_R^{TMPRSS2} = 0$. The ACE2 monomer has one glycosylated binding site. Here we derive the corresponding steric potential arising from the glycans trapped in the ACE2 binding site.

We start by considering the Kuhn length of the glycans, b_G; the unit size at which each unit can freely move, and orient in any direction. All details of chemical arrangements, bond rotation constraints, monomer-monomer interaction and monomer interaction with its local environment, are described by this parameter.

The mean squared end-to-end distance of the single glycan is given by

$$\langle r_0 \rangle^2 = N_G b_G^2, \tag{17}$$

while the length of a fully extended conformation is given by $r_{max} = N_G b_G$, where N_G is the number of monomers of the glycan. The probability that the end-to-end distance of a glycan is a given value, r, can be determined from a Gaussian distribution,

$$p(r, N_G) = \left(\frac{3}{2\pi N_G b_G^2}\right)^{\frac{3}{2}} exp\left(-\frac{3r^2}{2N_G b_G^2}\right). \tag{18}$$

Each branch can explore a hemispherical volume of radius r. Therefore, integrating equation (18) over this volume gives the partition function for a given branch,

$$q_j(r) = \int_0^{2\pi}\int_0^{\frac{\pi}{2}}\int_0^r \left(\frac{3}{2\pi N_G b_G^2}\right)^{\frac{3}{2}} exp\left(-\frac{3r^2}{2N_G b_G^2}\right) d\phi d\theta dr = \frac{3\pi}{4N_G b_G^2}\,\mathrm{erf}\left(\sqrt{\frac{3}{2N_G b_G^2}}r\right). \tag{19}$$

Hence the energy loss associated with the compression of a glycan with B branches upon binding is determined by the change in r from the average end-to-end distance $\langle r_0 \rangle$ to the binding distance $\langle r_B \rangle$,

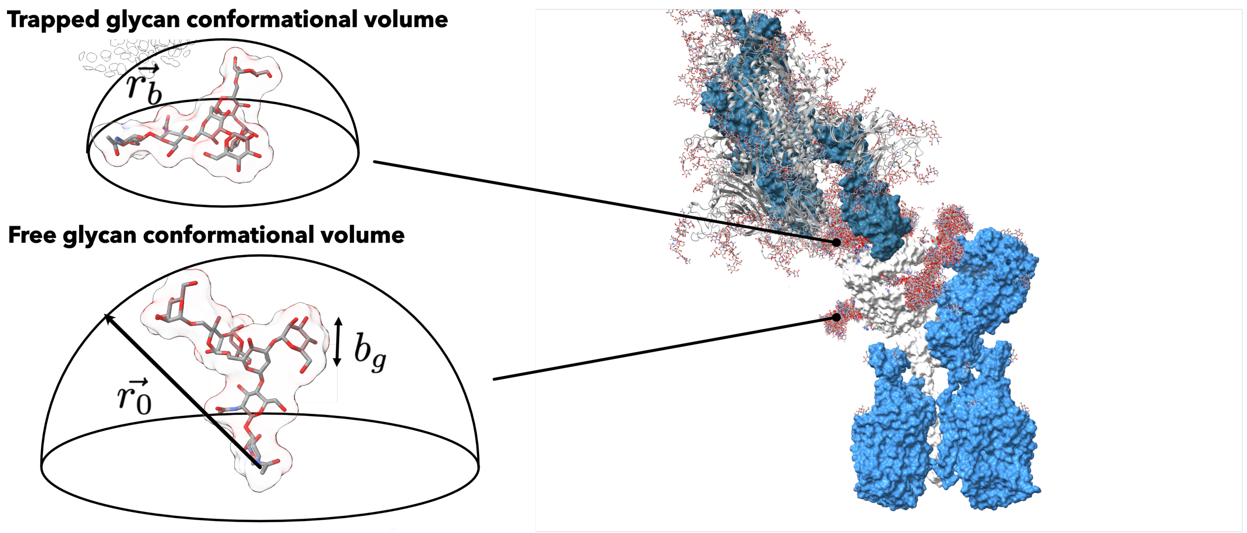

Figure S2: Entry receptor binding and glycans steric repulsion

$$U_{glycan} = \ln\left[\frac{\sum_{j=1}^{B} q_J(\langle r_0\rangle)}{\sum_{j=1}^{B} q_J(\langle r_B\rangle)}\right] = \ln\left[\sum_{j=1}^{B}\frac{\mathrm{erf}\left(\sqrt{\frac{3}{2}}\right)}{\mathrm{erf}\left(\sqrt{\frac{3}{2N_j b_G^2}}\langle r_B\rangle\right)}\right]. \tag{20}$$

The total free energy of repulsion can now be found by summing over each glycan, $u_R = \sum_{N_{Glycan}} U_{glycans}$, where N_{Glycan} is the number of glycans. Assuming that each glycan contains a similar amount of units, we can approximate

$$u_R = N_{Glycan} U_{glycan} = N_{Glycan} \ln\left[\sum_{j=1}^{B}\frac{\mathrm{erf}\left(\sqrt{\frac{3}{2}}\right)}{\mathrm{erf}\left(\sqrt{\frac{3}{2N_j b_G^2}}\langle r_B\rangle\right)}\right]. \tag{21}$$

	Figure 2	Figure 3	Figure 4a	Figure 4c	Figure 4d	Figure 4e	Figure 4f	Figure 5a	Figure 5b	Figure 5c
Viral radius , nm	50	50	50	50	50	50	50	50	50	
Viral titre , copies/ml	3.01E+07	6.02E+07	3.01E+07	3.01E+07	3.01E+07	3.01E+07	3.01E+07	6.02E+07	6.02E+07	6.02E+07
Number of Spikes	15	15	15	15	15	15	15	15	15	
Spike length, m	1.45E-08	1.45E-08	1.45E-08	1.45E-08	1.45E-08	1.45E-08	1.45E-08	1.45E-08	1.45E-08	1.45E-08
HS-Spike binding energy , kT	-16			-25,-18,-16	-25,-18,-16	-25,-18,-16	-25,-18,-16	-23	-23	-23
HS fraction of binding motif	0.1		0.1	0.1	0.1	0.1	0.1	0.1	0.1	0.1
HS chain length , m			1.3E-07	1.3E-07	1.3E-07	1.3E-07	1.3E-07			1.5E-07
HS number of chains , per μm^2					440	500	640			100
HS Kuhn Length , m	9E-09		9E-09	9E-09	9E-09	9E-09	9E-09			9E-09
ACE2 mean binding distance, m		2.7E-09						2.7E-09	2.7E-09	1E-09
ACE2 glycan branch length ,m								1.25E-09	1.25E-09	1.5E-09
ACE2 glycan number of branches								2	2	3
ACE2 number of trapped glycans		2						2	2	2
ACE2-Spike KD, M		2.06E-09						2.06E-09		2.06E-09
Number of ACE2 per μm^2		56							56	45
Number of ACE2 binding sites		3						3	3	3
Number of TMPRSS2 per μm^2		100							100	100
TMPRSS2-Spike KD, M		2.06E-09						2.06E-09		1.52E-08
Number of TMPRSS2 binding sites		1						1	1	1
Cell size, m2	1E-14	1E-14	1E-14					1E-14	1E-14	1E-14

Table 1: Model parameters fixed to generate the synthetic data.

References

[1] F. J. Martinez-Veracoechea and D. Frenkel, “Designing super selectivity in multivalent nano-particle binding,” *Proceedings of the National Academy of Sciences*, vol. 108, no. 27, pp. 10963–10968, 2011.

[2] C. Poirier, D. van Effenterre, B. Delord, L. Johannes, and D. Roux, “Specific adsorption of functionalized colloids at the surface of living cells: a quantitative kinetic analysis of the receptor-mediated binding.,” *Biochimica Et Biophysica Acta*, vol. 1778, p. 2450–2457, 2008.

[3] S. Hong, P. R. Leroueil, I. J. Majoros, B. G. Orr, J. R. B. Jr, and M. M. B. Holl, “The binding avidity of a nanoparticle-based multivalent targeted drug delivery platform.,” *Chemistry Biology*, vol. 14, pp. 107–115, 2007.

[4] X. Tian, S. Angioletti-Uberti, and G. Battaglia, “On the design of precision nanomedicines,” *Science Advances*, vol. 6, no. 4, 2020.

[5] M. Liu, A. Apriceno, M. Sipin, E. Scarpa, L. Rodriguez-Arco, A. Poma, G. Marchello, G. Battaglia, and S. Angioletti-Uberti, “Combinatorial entropy behaviour leads to range selective binding in ligand-receptor interactions.,” *Nature Communications*, vol. 11, p. 4836, 2020.

[6] S. Acosta-Gutiérrez, D. Matias, M. Avila-Olias, V. M. Gouveia, E. Scarpa, J. Forth, C. Contini, A. Duro-Castaño, L. Rizzello, and G. Battaglia, “A multiscale study of phosphorylcholine driven cellular phenotypic targeting.,” *ACS Central Science*, vol. 8, p. 891–904, 2022.

[7] J. Huskens, A. Mulder, T. Auletta, C. A. Nijhuis, M. J. W. Ludden, and D. N. Reinhoudt, “A model for describing the thermodynamics of multivalent host-guest interactions at interfaces.,” *Journal of the American Chemical Society*, vol. 126, p. 6784–6797, 2004.

[8] P. I. Kitov and D. R. Bundle, “On the nature of the multivalency effect: a thermodynamic model,” *Journal of the American Chemical Society*, vol. 125, no. 52, pp. 16271–16284, 2003.

[9] S. Angioletti-Uberti, “Exploiting receptor competition to enhance nanoparticle binding selectivity,” *Physical Review Letters*, vol. 118, no. 6, p. 068001, 2017.

[10] P. Varilly, S. Angioletti-Uberti, B. M. Mognetti, and D. Frenkel, “A general theory of dna-mediated and other valence-limited colloidal interactions,” *The Journal of Chemical Physics*, vol. 137, no. 9, p. 094108, 2012.
